# Bayesian methods for jointly estimating genomic breeding values of one continuous and one threshold trait

**DOI:** 10.1371/journal.pone.0175448

**Published:** 2017-04-14

**Authors:** Chonglong Wang, Xiujin Li, Rong Qian, Guosheng Su, Qin Zhang, Xiangdong Ding

**Affiliations:** 1 Department of Pig Genetics and Breeding, Institute of Animal Husbandry and Veterinary Medicine, Anhui Academy of Agricultural Sciences, Hefei, China; 2 Laboratory of Animal Genetics, Breeding and Reproduction, Ministry of Agriculture of China, National Engineering Laboratory for Animal Breeding, College of Animal Science and Technology, China Agricultural University, Beijing, China; 3 Center for Quantitative Genetics and Genomics, Department of Molecular Biology and Genetics, Aarhus University, Tjele, Denmark; 4 State Key Laboratory of Biocontrol, School of Life Sciences, Sun Yat-sen University, North Third Road, Guangzhou Higher Education Mega Center, Guangzhou, Guangdong, PR China; Fred Hutchinson Cancer Research Center, UNITED STATES

## Abstract

Genomic selection has become a useful tool for animal and plant breeding. Currently, genomic evaluation is usually carried out using a single-trait model. However, a multi-trait model has the advantage of using information on the correlated traits, leading to more accurate genomic prediction. To date, joint genomic prediction for a continuous and a threshold trait using a multi-trait model is scarce and needs more attention. Based on the previously proposed methods BayesCπ for single continuous trait and BayesTCπ for single threshold trait, we developed a novel method based on a linear-threshold model, i.e., LT-BayesCπ, for joint genomic prediction of a continuous trait and a threshold trait. Computing procedures of LT-BayesCπ using Markov Chain Monte Carlo algorithm were derived. A simulation study was performed to investigate the advantages of LT-BayesCπ over BayesCπ and BayesTCπ with regard to the accuracy of genomic prediction on both traits. Factors affecting the performance of LT-BayesCπ were addressed. The results showed that, in all scenarios, the accuracy of genomic prediction obtained from LT-BayesCπ was significantly increased for the threshold trait compared to that from single trait prediction using BayesTCπ, while the accuracy for the continuous trait was comparable with that from single trait prediction using BayesCπ. The proposed LT-BayesCπ could be a method of choice for joint genomic prediction of one continuous and one threshold trait.

## Introduction

With the developments of single-nucleotide polymorphism (SNP) chips and genotyping by sequencing, a huge number of genome-wide polymorphisms have been widely used in practical animal and plant breeding programs. Genomic selection (GS) can use the information of genome-wide markers to accurately predict the genetic merit of an animal without the need of its own phenotypic information[1]. In a typical process of genomic prediction, SNP effects are estimated using a training population consisting of individuals with both SNP genotypes and phenotypes, and then these estimated effects are used to build a prediction equation to calculate the genomic estimated breeding values (GEBVs) for breeding candidates, based on their SNP genotypes. Therefore, an appropriate model is a key to accurately predict GEBVs in GS.

Many Bayesian models have been proposed to estimate GEBVs. In the first paper of genomic selection[1], two Bayesian methods (BayesA and BayesB) were presented to estimate genomic breeding values and have been extensively used in the subsequent studies of genomic selection[2–5]. However, there are two drawbacks of BayesA and BayesB. One drawback is that the full-conditional posterior distribution of a locus-specific variance has only one additional degree of freedom compared to its prior distribution regardless of the number of genotypes or phenotypes, and the shrinkage of SNP effects depends strongly on the scale parameter $S_{a}^{2}$ as pointed out by Gianola et al.[6]. The other one is that for BayesB the possibility π of a SNP having zero effect should be given as known. To overcome these two drawbacks, Habier et et al.[7] proposed two new methods, BayesCπ and BayesDπ. For BayesCπ, a single variance is common to SNPs having non-zero effects instead of locus-specific variances, while for BayesDπ, the scale parameter, $S_{a}^{2}$, for the scaled inverse chi-square prior of the locus-specific variance is treated as an unknown with a Gamma(1,1) prior. Both BayesCπ and BayesDπ treat π as an unknown, and need to be inferred from the data.

Wang et al.[8] extended the three Bayesian methods (BayesA, BayesB, and BayesCπ) for a Gaussian trait to be used for a threshold trait. The extended methods are correspondingly termed BayesTA, BayesTB and BayesTCπ, respectively. From the results of a simulation study on a threshold trait, the three BayesT methods performed better than the corresponding Bayesian methods when treating a threshold trait as a Gaussian trait, and BayesTCπ performed the best among the three new methods, so it was recommended as the method of choice for threshold traits in genomic selection[8]. In addition, a Bayesian multi-locus association mapping for threshold traits using a threshold model was also proposed by Iwata et al.[9], and their approach could reduce both false-positive and false-negative rates in detecting QTL to reasonable levels.

Currently, a single trait model is usually applied in practical genomic prediction. Theoretically, a joint genomic evaluation of genetically correlated traits should lead to more accurate predictions than single trait genomic prediction as shown in some studies[10–14]. However, these studies all focused on multiple continuous traits, and studies on a joint genomic prediction of continuous and discrete traits are scarce so far. In practical animal breeding, people often need to make selection for continuous and categorical traits simultaneously (e.g., birth weight and calving ease in cattle and growth rate and leg weakness in pig). In conventional breeding value prediction based on phenotypic and pedigree information, methods for joint analysis of continuous and threshold traits have been well established[15–18]. In this study, we proposed a BayesCπ bivariate model for a joint genomic prediction of one normal distributed trait and one threshold trait, which was termed as LT-BayesCπ. We validated LT-BayesCπ with simulated data and with a common data set from the 14^th^ QTL-MAS workshop[19]. The accuracy of genomic prediction obtained from LT-BayesCπ was compared with that from BayesCπ or BayesTCπ based on single-trait model. Furthermore, factors affecting the performance of LT-BayesCπ were investigated as well.

## Methods

### Models

Let $y_{1}^{\prime }=\{y_{1,i}\}$ (*i* = 1, 2,…, *n*) be the vector of observations for a continuous trait, $y_{2}^{\prime }=\{y_{2,i}\}$ (*i* = 1, 2,…, *n*) be the vector of observations for a threshold trait and **l**′ = {*l*_*i*_} (*i* = 1, 2,…, *n*) be the vector of underlying latent variables or liabilities associated with the threshold trait. The linear-threshold model is

$$\left[\begin{array}{c} y_{1}\\ l \end{array}\right]=\left[\begin{array}{cc} X_{1} & 0\\ 0 & X_{2} \end{array}\right]\left[\begin{array}{c} \beta_{1}\\ \beta_{2} \end{array}\right]+\left[\begin{array}{cc} Z & 0\\ 0 & Z \end{array}\right]\left[\begin{array}{c} g_{1}\\ g_{2} \end{array}\right]+\left[\begin{array}{c} e_{1}\\ e_{2} \end{array}\right],$$

where ***β***_**1**(**2**)_ is a vector of fixed effects, ***g***_**1**(**2**)_ is the vector of SNP effects, ***e***_**1**(**2**)_ is the vector of the random residuals, ***X***_**1**(**2**)_ is the incidence matrix for ***β***_**1**(**2**)_, and **Z** is the matrix of genotype indicators with values 0,1 or 2 for genotypes 11, 12 and 22, respectively. Let $v^{\prime }=[y_{1}^{\prime },l\prime ]$. It is assumed that, given **β** and **g**, **v** is distributed as

$$v|\beta ,g,R_{e}∼N([\begin{array}{c} X_{1}\beta_{1}+Zg_{1}\\ X_{2}\beta_{2}+Zg_{2} \end{array}],R),$$

where $\beta^{\prime }=[\beta_{1}^{\prime },\beta_{2}^{\prime }]$, $g^{\prime }=[g_{1}^{\prime },g_{2}^{\prime }]$, and **R** = ***R***_***e***_ ⊗ **I** with $R_{e}=\left[\begin{array}{cc} \sigma_{e_{1}}^{2} & \sigma_{e_{1,2}}\\ \sigma_{e_{1,2}} & \sigma_{e_{2}}^{2} \end{array}\right]$.

Then, given **β**, **g** and ***R***_***e***_, the sampling model can be written as

$$p(v|\beta ,g,R_{e})\propto {\left|R_{e}\right|}^{-\frac{n}{2}}exp[-\frac{1}{2}tr(R_{e}^{-1}S_{e})]$$

where $S_{e}=\left[\begin{array}{cc} e_{1}^{\prime }e_{1} & e_{1}^{\prime }e_{2}\\ e_{2}^{\prime }e_{1} & e_{2}^{\prime }e_{2} \end{array}\right]$.

### MCMC implementation of LT-BayesCπ

#### Prior distributions

In this study, the following prior distributions are assumed for building a hierarchical model.

For fixed effects **β**:

$$\beta |\beta_{min},\beta_{max}∼\cup (\beta_{min},\beta_{max})$$

For SNP effects **g**:

Each SNP has either a zero effect for both traits or non-zero effect for at least one trait with probabilities π and 1−π, respectively. For the latter case, its prior distribution is bivariate normal, i.e.,

$$\left[\begin{array}{c} g_{1}\\ g_{2} \end{array}\right]|G_{0}∼N([\begin{array}{c} 0\\ 0 \end{array}],G_{0}⊗I),$$

where $G_{0}=\left[\begin{array}{cc} \sigma_{g1}^{2} & \sigma_{g1,2}\\ \sigma_{g1,2} & \sigma_{g2}^{2} \end{array}\right]$.

For ***G***_**0**_ and ***R***_***e***_

***G***_**0**_ and ***R***_***e***_ are assumed to follow inverse Wishart distributions:

$$p(G_{0}|v_{g},V_{g})\propto {\left|G_{0}\right|}^{-\frac{1}{2}(v_{g}+3)}\exp\left[-\frac{1}{2}tr(G_{0}^{-1}V_{g}^{-1})\right],$$

$$p(R_{e}|,V_{e})\propto {\left|R_{e}\right|}^{-\frac{1}{2}(v_{e}+3)}\exp\left[-\frac{1}{2}tr(R_{e}^{-1}V_{e}^{-1})\right],$$

where *v*_*i*_ and ***V***_*i*_ (*i* = *g*, *e*) are the usual hyper-parameters of the scaled inverse Wishart distribution, which are assumed to be known. In particular, if *v*_*i*_ = -3 and ***V***_*i*_ = **0**, these two distributions reduce to improper uniform distributions [17].

For the thresholds **t**:

Suppose the threshold trait consists of *k* categories, then there are *k*-1 hypothetical thresholds (**t***’* = [*t*_1_, *t*_2_, …, *t*_k-1_]) in the underling latent scale. These thresholds are assumed to follow ordered uniform distribution in the interval [*t*_min_, *t*_max_]. However, two of the thresholds must be fixed, so as to ensure the identifiability of the parameters. Typical choices are *t*_1_ = 0 and *t*_2_ = 1[17]. Therefore, there are *k*-3 unknown thresholds. The joint prior density of **t** is

$$p(t)=(k-3)!{\left(\frac{1}{t_{max}-t_{min}}\right)}^{k-3}I(t\in T),$$

where *T* = {(*t*_1_ = 0,*t*_2_ = 1,*t*_2_,…,*t*_*k*−1_)|*t*_*min*_ ≤ *t*_1_ ≤ ⋯ ≤ *t*_*k*−1_ ≤ *t*_*max*_}, I is an indicator vector. If t∈T, elements of I are 1, otherwise, elements of I are 0. In this study, we set *t*_max_ and *t*_min_ as μ±10σ based on the normal distribution of liability.

For the probability of zero effect π:

p(π)∼∪(0,1).

#### Joint posterior distribution

The parameter vector is augmented with the unobserved liabilities **l** for the threshold trait and with the indicator variables **δ** for SNP effects (indicating whether a SNP has an effect on the traits (with probability of 1 -π) or not (with probability of π)), and is represented as (**Ω**,**l**,**δ**), where **Ω** = (**β**,**g**,***G***_**0**_,***R***_***e***_,**t**,***π***).

The joint posterior distribution of (**Ω**,**l**,**δ**) is

$$p(\Omega ,l,\delta |y_{1},y_{2},\sigma_{e2}^{2}=1,t_{1}=0)\propto p(y_{1},y_{2}|\Omega ,l,\delta )p(\Omega ,l,\delta )=p(y_{1},l|\Omega )p(y_{2}|\Omega ,l)p(\Omega ,\delta )$$

#### Fully conditional posterior distributions

**Liabilities.** The fully conditional posterior distribution of liability *l*_*i*_ is a truncated normal distribution within the range from *t*_*j*−1_ to *t*_*j*_, i.e, *l*_*i*_|*ELSE* ∼ *N*(E(*l*_*i*_|ELSE), Var(*l*_*i*_|ELSE)) and truncated within *t*_*j*−1_ to *t*_*j*_ with

$$E(l_{i}|ELSE)=x_{2,i}^{\prime }\beta_{2}+z_{2,i}^{\prime }g_{2}+\frac{\sigma_{e1,2}}{\sigma_{e1}^{2}}(y_{1,i}-x_{1,i}^{\prime }\beta_{1}-z_{1,i}^{\prime }g_{1})$$

and

$$Var(l_{i}|ELSE)=\sigma_{e2}^{2}(1-\frac{{\left(\sigma_{e1,2}\right)}^{2}}{\sigma_{e1}^{2}\sigma_{e2}^{2}})$$

**Location parameters.** Write the mixed model equations (MME) as $C\hat{\theta }=r$, where **C** is left hand, ***r*** is right hand, and $\hat{\theta }$ is a vector of unknown parameters, $\hat{\theta }=\left[\begin{array}{c} \beta \\ g \end{array}\right]$. The fully conditional posterior distribution of *θ*_*i*_ is

$$\theta_{i}|ELSE∼N(\tilde{\theta_{i}},C_{ii}^{-1}),$$

where $\tilde{\theta_{i}}=C_{i,i}^{-1}(r_{i}-C_{i,-i}\theta_{-i})$.

**Dispersion parameters.** The fully conditional posterior distributions of the co-variance matrix of SNP effects and residual effects are

$$G_{0}|ELSE∼{IW}_{2}({\left(V_{g}^{-1}+S_{g}\right)}^{-1},v_{g}+q)$$

$$R_{e}|ELSE∼{IW}_{2}({\left(V_{e}^{-1}+S_{e}\right)}^{-1},v_{e}+n)$$

If the prior distributions of **G**_0_ and **R**_*e*_ are flat (in case of *v*_*i*_ = -3 and ***V***_***i***_ = **0,** *i* = *g*, *e***)**, these two distributions reduce to

$$G_{0}|ELSE∼{IW}_{2}(S_{g}^{-1},q-3)$$

$$R_{e}|ELSE∼{IW}_{2}(S_{e}^{-1},n-3)$$

The fully posterior distributions of other parameters and the procedure of the Gibbs sampler are similar to single trait BayesTCπ which was described by Wang et al.[8].

It should be noted that when the categorical trait is binary, i.e., it has only two categories with one threshold, it is not possible to fix two thresholds. In this case, a usual parameterization is to fix the residual variance of the binary trait ($\sigma_{e_{2}}^{2}$) to be 1 and the threshold to be 0. With this parameterization, rather than adopting an inverse Wishart prior for ***R***_*e*_, one can assign a conditional inverse Wishart prior (conditional on $\sigma_{e_{2}}^{2}=1$) and the fully conditional posterior distribution of ***R***_e_ is conditional scaled inverted Wishart given $\sigma_{e_{2}}^{2}=1$ [17]. A general algorithm for drawing samples from such a distribution was proposed by Korsgaard et al. (1999) [20] (see also [17]). Following their algorithm, the realized values from the distribution $R_{e}|ELSE,\sigma_{e2}^{2}=1$ can be obtained in the following way:

Let $V=S_{e}^{-1}=\left[\begin{array}{cc} V_{11} & V_{12}\\ V_{21} & V_{22} \end{array}\right]$
Sample *x*_1_ from *W*_1_(*V*_11_,*n*−3).
Sample *x*_2_ from $N(V_{11}^{-1}V_{12},x_{1}^{-1}V_{22.1})$, where $V_{22.1}=V_{22}-V_{12}^{2}V_{11}^{-1}$.
Let $T_{11}=x_{1}^{-1}+x_{2}^{2}$, *T*_12_ = −*x*_2_, and *T*_22_ = 1, then *T*_11_, *T*_12_ and *T*_22_ are the realized values from the distribution $R_{e}|ELSE,\sigma_{e2}^{2}=1$.

### Simulation study

#### Data simulation

To evaluate the proposed method LT-BayesCπ, we carried out a series of simulations using the multiple-trait genomic simulation software GPOPSIM [21]. For simplicity, we simulated a continuous and a binary threshold trait with different genetic correlation levels between them.

Briefly, the simulation started with a base population consisting of 100 individuals, followed by 1,000 non-overlapping historical generations with the same population size in each generation, denoted as generation -999 to generation 0. In each historical generation, 50 males were randomly mated with 50 females and each mating produced one male and one female offspring. All markers were monomorphic at the starting status in the base population, then polymorphic markers were generated in the following generations by mutation with a mutation rate of 1.25 × 10^−3^, and reached the mutation-drift equilibrium status through genetic drift. After 1,000 historical generations, six generations, numbered from 1 to 6, were further generated. In generation 1, the population size was expanded from 100 to 1,000 by increasing the number of offspring of each female in generation 0 from 2 to 20 (10 males and 10 females). From generation 1 to 5, 50 males were randomly selected from the 500 males to be sires of the next generation and all 500 females were used as dams. Each selected male mated randomly with 10 females and each female produced two offspring (one male and one female). Generations 1 and 2 were treated as training population across the scenarios studied, and generations 3–6 were validation (candidate) populations.

We simulated five chromosomes with a total length of 5 Morgan (1 Morgan per chromosome). On each chromosome, 2,000 markers were evenly distributed and every two adjacent loci were assumed to harbor a potential QTL. The final true QTL were randomly sampled from these potential QTL. Based on the distance between two adjacent loci, Haldane’s mapping function was used to calculate the probability of recombination between adjacent loci.

Two genetically correlated traits (denoted as Trait A and Trait B) were simulated. Trait A was a continuous trait with a normal distribution, and Trait B was a binary threshold trait with normally distributed underlying liabilities. The sampled true QTL were divided into three groups, Group1, Group2 and Group3. QTL in Group1 had pleiotropic effects on both traits, and QTL in Group2 and Group3 had effects on trait A only or trait B only, respectively. The allele substitution effects of each QTL in Group1 were sampled from a bivariate normal distribution with varied genetic correlation (*r*_*AB*_) between the two traits and those in Group2 or Group3 were drawn from univariate normal distributions, see details in[14]. For any *r*_*AB*_ between traits A and B, we set the ratio of QTL in Group1, Group2 and Group3 as 0.8:0.1:0.1, except for the case of *r*_*AB*_ = 0, where the ratio of QTL in Group1, Group2 and Group3 was set as 0.0:0.5:0.5. The allele substitution effects were re-scaled to ensure that the total additive genetic variances of trait A and B were equal to 2.0 and 1.0, respectively. The environmental correlation was assumed to be 0.0, and the environmental effects on the two traits were sampled independently from univariate normal distributions.

True breeding values (TBV), which were generated by summing effects of all QTL, were added to environmental effects to produce phenotypic values of trait A and liability values of trait B. Genotypes and TBV were simulated for all individuals from generations 1 to 6, but phenotypic or liability values were only assigned to the 2,000 individuals in generations 1 and 2 (training population). For trait B, a threshold value was assigned according to the assumed incidence, and the observed phenotype of an individual with liability value lower than the threshold value was 0, otherwise it was 1.

We firstly simulated a standard scenario in which the following parameters were assigned: heritabilities of the two traits: $h_{A}^{2}=0.3$ and $h_{B}^{2}=0.1$, number of QTL = 60, genetic correlation between traits A and B: *r*_*AB*_ = 0.50, and the incidence (individuals with observation 1) for trait B = 0.30. To investigate the impacts of various factors on genomic prediction, alternative scenarios were generated by using one of the following parameters to replace the corresponding parameter in the standard scenario. These alternative parameters were: genetic correlation *r*_*AB*_ (0.00, 0.20, and 0.80), number of QTL (20, 200, and 500), heritability of the continuous trait A ($h_{A}^{2}$ = 0.5 and 0.8), heritability of the binary threshold trait B ($h_{A}^{2}$ = 0.3 and 0.5), and incidences of trait B (0.05, 0.1, and 0.5). For each scenario, 20 replicated datasets were simulated.

#### Data from the 14^th^ QTL-MAS workshop

The common data set of the 14^th^ QTL-MAS workshop[19] was also used to evaluate the proposed method LT-BayesCπ. This data set contains 3,226 individuals in five generations (F0-F4). All individuals have genotypes, and only 2,326 individuals in generations F0-F3 have phenotypic records on two traits: a quantitative trait Q and a binary threshold trait B. Individuals with phenotypic records (F0-F3) were treated as training population and those without phenotypic records (F4) as validation (candidate) population. Five autosomal chromosomes were simulated, each 100Mbp long, and contained 10,031 biallelic SNP without any missing data and genotype errors. The quantitative trait Q was controlled by 37 QTL (30 additive QTL, 4 epistatic QTL and 3 imprinted QTL). Out of the 30 additive QTL, 22 also influenced the binary threshold trait B which was not controlled by any other QTL. The narrow-sense heritability (h^2^) for trait Q was 0.52 for males and 0.39 for females, whereas *h*^2^ for trait B was 0.48 for both males and females. The correlation between breeding values for the two traits was 0.59 for males and 0.68 for females.

### Estimation of SNP effects

Three Bayesian methods were implemented to estimate SNP effects based on the training population. The proposed new method LT-BayesCπ was used for the joint analysis of both traits, while BayesCπ was used for the continuous trait and BayesTCπ for the threshold trait. For each model, the Markov chains were run for 50, 000 cycles of Gibbs sampling, and the first 30, 000 cycles were discarded as burn-in. All remaining samples of SNP effects after burn-in were averaged to obtain the estimates of SNP effects.

In the analysis of both simulated data sets by LT-BayesCπ, we assumed the values of the hyper-parameters *v*_*g*_, **V**_g_, *v*_*e*_ and **V**_e_ of the prior distributions of **G**_0_ and **R**_e_ to be -3, **0**, -3, and **0**, respectively, such that they reduced to flat priors. In addition, in both simulated data sets, the threshold trait was binary. So, as mentioned above, we fixed the threshold to be 0 and the residual variance of the binary trait to be 1 in the analysis. We drew samples of the fully conditional posterior distribution of **R**_e_ using the algorithm mentioned above.

### Accuracy of genomic prediction

GEBVs for individuals in the candidate population were calculated as the sum of all marker effects according to their marker genotypes. For each trait, accuracy was measured as the correlation between TBV and GEBV (*r*_*TBV*,*GEBV*_), and the regression of TBV on GEBV (*b*_*TBV*,*GEBV*_) was also calculated for assessing the bias of genomic prediction. However, for the binary trait, the scale of GEBV was not the same as TBV due to the restriction of $\sigma_{e2}^{2}=1$. Thus, *b*_*TBV*,*GEBV*_ must be rescaled back to the original scale by using $b_{TBV,GEBV}/\sqrt{v_{e2}}$, where *v*_*e*2_ is the true residual variance of the liabilities in the simulation. In addition, a t-test was carried out to investigate the differences in accuracy obtained from LT-BayesCπ and the single-trait methods BayesCπ or BayesTCπ.

## Results

### Analysis of simulated data

#### Estimates of SNP effects in the standard scenario

**Fig 1:**
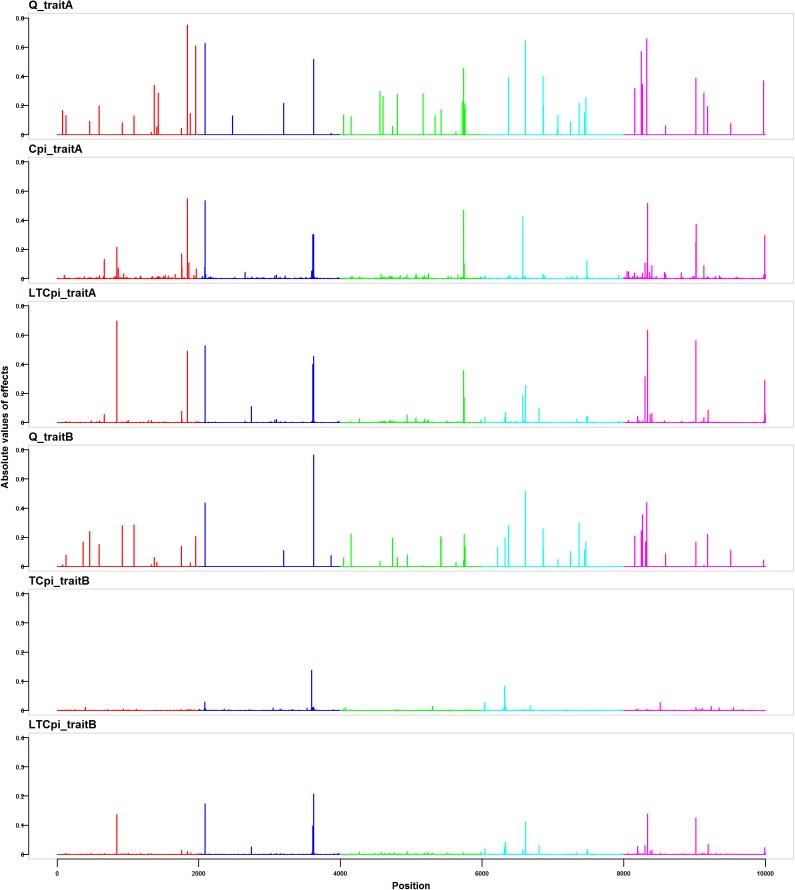
**Simulated QTL effects and estimated SNP effects for the continuous trait (trait A) and the binary threshold trait (trait B) from a randomly selected replicate in the standard scenario.** Panels Q_traitA and Q_traitB show the absolute values of the simulated true QTL effects. Panels Cpi_traitA, LTCpi_traitA, TCpi_traitB, and LTCpi_traitB show the absolute values of estimated SNP effects by BayesCπ for trait A, LT-BayesCπ for trait A, BayesTCπ for trait B, and LT-BayesCπ for trait B, respectively.

Fig 1 shows the simulated QTL effects and the estimated SNP effects by LT-BayesCπ, BayesCπ and BayesTCπ from a randomly selected replicate in the standard scenario. For the continuous trait (Trait A), the simulated absolute SNP effects ranged from 0–0.75, and the estimated absolute SNP effects ranged from 0–0.55 from BayesCπ and 0–0.70 from LT-BayesCπ, respetively. For the binary threshold trait (Trait B), the simulated absolute SNP effects ranged from 0–0.77, and the estimated absolute SNP effects ranged from 0–0.14 from BayesTCπ and 0–0.21 from LT-BayesCπ, respectively. Most of the segments containing QTL with large effects were mapped by all methods.

#### Accuracies of GEBVs in the standard scenario

Table 1 shows accuracies of GEBVs in terms of correlations between GEBVs and simulated true breeding values in generations 3–6 (candidate population) in the standard scenario. For all methods, accuracies of genomic prediction declined with generations as expected; the only exception is that the accuracy for trait B obtained from LT-BayesCπ in generation 5 is slight lower than that in generation 6, probably due to sampling error. For trait B, LT-BayesCπ performed much better than BayesTCπ consistently in all generations, and was about 11 percent higher in each generation (P<0.001). However, for trait A, no improvement was obtained from LT-BayesCπ compared with BayesCπ in all generations (P>0.10).

**Table 1:** Accuracies of GEBVs (mean±s.e. from 20 replicates) obtained from three methods in generations 3–6 in the standard scenario.

Method	Trait	Generation 3	Generation 4	Generation 5	Generation 6
**BayesCπ**	A	0.771±0.010	0.740±0.012	0.717±0.013	0.714±0.012
**LT-BayesCπ**	A	0.761±0.010	0.728±0.011	0.703±0.013	0.699±0.012
**BayesTCπ**	B	0.465±0.023	0.420±0.022	0.397±0.025	0.395±0.024
**LT-BayesCπ**	B	0.581±0.020	0.533±0.022	0.510±0.022	0.522±0.023

#### Impact of genetic correlation

Table 2 shows the accuracies of genomic prediction in generation 3 under different genetic correlations (0, 0.20, 0.50 and 0.80), while keeping the other parameters the same as in the standard scenario. For trait A, the accuracies of GEBV from LT-BayesCπ were nearly equal to those from BayesCπ regardless of the genetic correlation. It suggested that LT-BayesCπ performs comparably with BayesCπ for continuous traits. We also observed that differences in accuracies between LT-BayesCπ and BayesCπ were slightly decreased with increasing the genetic correlation between two traits. For trait B, in the case of no genetic correlation between the two traits, the accuracy of genomic prediction from LT-BayesCπ was slightly lower than that from BayesTCπ. However, the accuracies of genomic prediction obtained from LT-BayesCπ were dramatically increased with the increase of the genetic correlation. The improvement in the accuracy of LT-BayesCπ over BayesTCπ was consistently increased with the increase of the genetic correlation.

**Table 2:** Accuracies of GEBVs for the two traits in generation 3 in four scenarios of different genetic correlations.

Genetic correlation	Method	Accuracy	
		Trait A	Trait B
**0.00**	BayesCπ/TCπ	0.817±0.008	0.545±0.024
	LT-BayesCπ	0.800±0.010	0.492±0.026
	Increment	-0.017	-0.053
**0.20**	BayesCπ/TCπ	0.780±0.010	0.479±0.019
	LT-BayesCπ	0.770±0.010	0.525±0.017
	Increment	-0.010	0.046
**0.50**	BayesCπ/TCπ	0.771±0.010	0.465±0.023
	LT-BayesCπ	0.761±0.010	0.581±0.020
	Increment	-0.010	0.116^***^
**0.80**	BayesCπ/TCπ	0.762±0.008	0.473±0.019
	LT-BayesCπ	0.756±0.008	0.674±0.016
	Increment	-0.006	0.201^***^

*** P-value < 0.001

** P-value < 0.01

*P-value < 0.05

Besides the genomic breeding values, we also estimated the genetic and residual correlations between the continuous and the binary traits and the proportions of true QTL (π). As shown in Table 3, the estimates of π obtained by LT-BayesCπ were very close to the assigned 0.006 (60 QTL) in all scenarios with different genetic correlations. Meanwhile, the estimates of genetic and residual correlations were almost unbiased in all cases, except in the case of genetic correlation of 0.80, where the estimate of genetic correlation was biased downwards.

**Table 3:** The estimated genetic correlations ($\hat{r_{g}}$), residual correlations $\left(\hat{r_{e}}\right)$, and proportions of true QTL ($\hat{\pi }$) from LT-BayesCπ in four scenarios of different genetic correlations.

Genetic correlation	$\hat{r_{g}}$	$\hat{r_{e}}$	$\hat{\pi }$
**0.00**	0.026±0.030	-0.011±0.007	0.0051±0.0004
**0.20**	0.178±0.036	-0.006±0.007	0.0068±0.0003
**0.50**	0.471±0.036	0.005±0.008	0.0061±0.005
**0.80**	0.674±0.024	0.020±0.006	0.0060±0.0003

The assigned r_e_ and π are 0 and 0.006, respectively.

The regression coefficients of the simulated TBV on GEBV are presented in Table 4. For trait A, the regression coefficients from BayesCπ were all slightly lower than 1, while those from LT-BayesCπ were slightly greater than 1. For trait B, because the scale of GEBV is not the same as TBV due to the restriction of $\sigma_{e2}^{2}=1$, the regression coefficients must be rescaled back to original scale. After rescaling, both LT-BayesCπ and BayesTCπ generated nearly unbiased genomic prediction, i.e., the regression coefficients were closer to 1.0 in all situations except for LT-BayesCπ in the scenario of genetic correlation = 0.

**Table 4:** Regression coefficients of TBVs on GEBVs in generation 3 in four scenarios with different genetic correlations.

Genetic correlation	Method	Regression coefficient	
		Trait A	Trait B*
**0.00**	BayesCπ/TCπ	0.996±0.018	0.962±0.062
	LT-BayesCπ	1.169±0.023	0.787±0.046
**0.20**	BayesCπ/TCπ	0.988±0.011	1.176±0.168
	LT-BayesCπ	1.153±0.014	0.874±0.047
**0.50**	BayesCπ/TCπ	0.982±0.017	1.093±0.156
	LT-BayesCπ	1.134±0.022	0.864±0.035
**0.80**	BayesCπ/TCπ	0.976±0.016	1.124±0.152
	LT-BayesCπ	1.140±0.023	0.918±0.038

* Rescaled regression coefficients of TBVs on GEBVs

#### Impact of number of QTL

**Fig 2:**
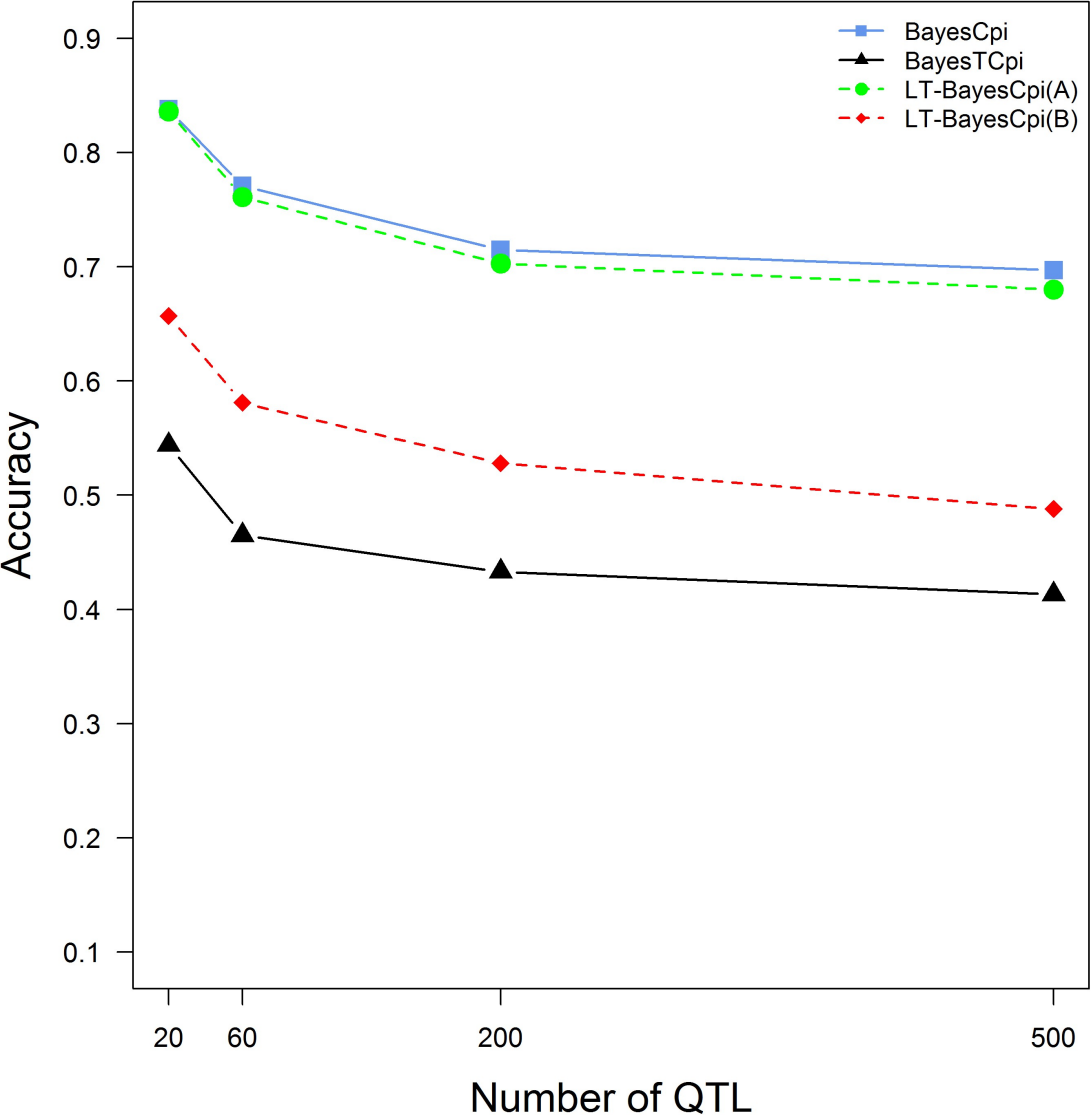
Accuracies of GEBVs from three methods in generation 3 when the number of simulated true QTL changed from 20 to 500.

As shown in Fig 2, BayesCπ, BayesTCπ and LT-BayesCπ all were sensitive to the number of QTL affecting traits of interest, and the accuracies of genomic prediction from them decreased rapidly with the increase of the number of QTL. When the number of QTL increased from 20 to 500, the accuracies of GEBVs were decreased by 0.141, 0.131, 0.156 and 0.169 for BayesCπ, BayesTCπ, LT-BayesCπ (Trait A) and LT-BayesCπ (Trait B), respectively. In the same scenario, LT-BayesCπ was equivalent to single trait method BayesCπ for the continuous trait A and performed better than BayesTCπ for the threshold trait B. For trait B, the accuracies from LT-BayesCπ were 0.113, 0.116, 0.095, 0.075 higher (P <0.001) than those from BayesTCπ in cases with 20, 60, 200 and 500 QTL, respectively.

#### Impact of heritability

**Fig 3:**
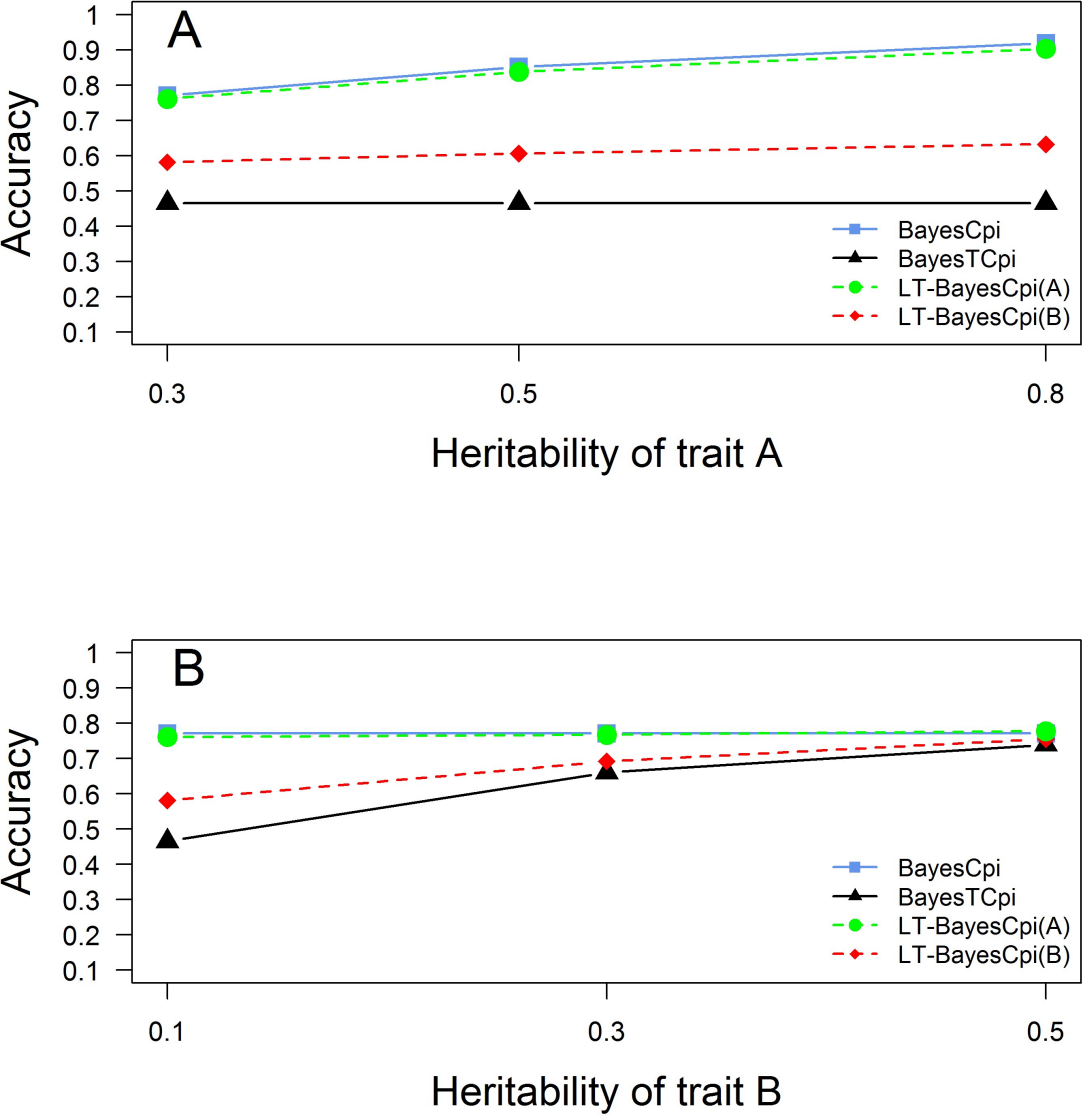
Accuracies of GEBVs from three methods in generation 3 with different heritabilities. **A:** heritability of the continuous trait A changing from 0.3 to 0.8, while keeping the heritability of the binary threshold trait constant (0.1); **B:** heritability of the binary threshold trait B changing from 0.1 to 0.5, while keeping the heritability of the continuous trait constant (0.3).

Fig 3 shows the accuracies of GEBVs from the three methods in generation 3 under different heritabilities of one trait, while keeping the other parameters the same as in the standard scenario. By increasing the heritability of trait A from 0.3 to 0.8 and keeping the heritability of trait B unchanged, the accuracies of GEBVs for trait A from BayesCπ and LT-BayesCπ consistently increased as expected. In addition, for trait B, the accuracies from LT-BayesCπ also increased from 0.581 to 0.632 (the accuracy from BayesTCπ was 0.465). On the other hand, by increasing the heritability of trait B from 0.1 to 0.5 and keeping the heritability of trait A unchanged, the accuracies of GEBVs for trait B from BayesTCπ and LT-BayesCπ consistently increased as expected. In addition, for trait A, the accuracies from LT-BayesCπ also increased from 0.761 to 0.777 (the accuracy from BayesCπ was 0.771). These results indicate that increasing heritability of one trait is helpful to improve the accuracy of genomic prediction of the genetic correlated traits when jointly analyzing them. It should be noted that, for trait A, when the heritability of the continuous trait A was 0.3 and the heritability of the threshold trait B was 0.5, the accuracies from LT-BayesCπ was slightly higher than BayesCπ, while in all other cases, they were slightly lower.

#### Impact of incidence of the threshold trait

**Fig 4:**
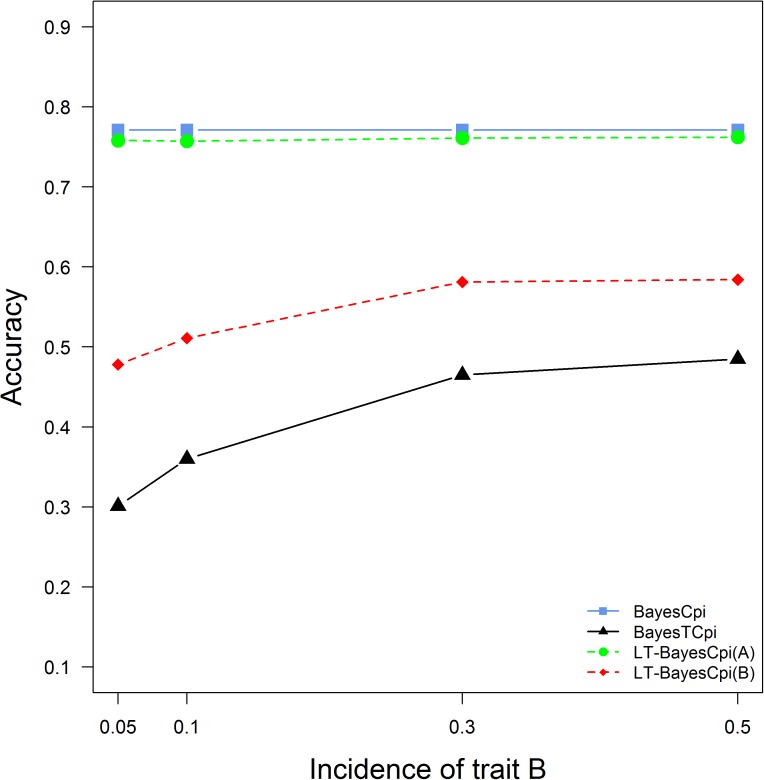
Accuracies of GEBVs from three methods in generation 3 when the incidence of the binary threshold trait increased from 0.05 to 0.5.

The accuracies of GEBVs for different incidences of the threshold trait B are presented in Fig 4 (the other parameters were the same as in the standard scenario). With increased incidence from 5% to 50%, the accuracies of GEBVs for trait B from both BayesTCπ and LT-BayesCπ increased consistently as expected. However, the superiority of LT-BayesCπ over BayesTCπ decreased as the incidence increased. The accuracies of genomic prediction for trait A from LT-BayesCπ were not influenced by the incidence of trait B.

### Analysis of the common dataset of the 14^th^ QTL-MAS workshop

For each trait and each Bayesian method, the accuracy and bias of GEBVs in the candidate population are shown in Table 5. For the quantitative trait Q, again the accuracy of genomic prediction from LT-BayesCπ was comparable with that from BayesCπ as they did in our simulation study. For the threshold trait B, LT-BayesCπ generated higher accuracy and less bias of GEBV than BayesTCπ, which was consistent with the results from the analysis of the simulated data as well.

**Table 5:** Accuracies and bias of GEBVs from three methods for the common dataset from the 14^th^ QTL-MAS workshop.

Trait	Methods	Accuracy	Regression coefficient
**Q**	BayesCπ	0.677	0.955
	LT-BayesCπ	0.681	0.933
**B**	BayesTCπ	0.829	1.228^*^
	LT-BayesCπ	0.867	1.055^*^

* Rescaled regression coefficients, i.e., $b_{TBV,GEBV}/\sqrt{v_{e2}}$, where *v*_*e*2_ = 18.16 is the true residual variance for the threshold trait B in the simulation.

## Discussion

In traditional genetic evaluation, a multiple-trait model was proved to be able to increase the accuracy of the estimated breeding values by making use of information from genetically correlated traits[22], and has been widely implemented in practical breeding value estimation. Since the concept of genomic selection was proposed in 2001[1], many models, such as GBLUP, BayesA, BayesB, BayesCπ, have been developed for genomic prediction, and most studies focused on genomic prediction for a single continuous trait. A few extensions of BayesA, BayesB and BayesCπ were proposed for a single threshold trait recently [8,23,24]. Recently, some investigations took the correlation structure between traits into account for joint genomic prediction of multiple continuous traits, showing increased accuracy of genomic prediction[10–14]. However, researches on joint genomic prediction of continuous traits and threshold/binary traits are still scarce. In this study, we developed a novel method, LT-BayesCπ, to deal with the joint genomic prediction of one continuous and one threshold trait that are genetically correlated. The results from our simulation study and the common dataset of the 14^th^ QTL-MAS workshop indicated that, in all scenarios considered, when analyzing a continuous and a binary trait jointly using LT-BayesCπ and, both the accuracy and the unbiasedness of GEBV for the binary trait could be remarkably improved in comparison with that from single trait analysis using BayesTCπ, while for the continuous trait the accuracy and the unbiasedness were comparable with that from single trait analysis using BayesCπ.

Genetic correlation between traits is essential for getting benefit from multiple trait analysis. Genetic correlation between two traits arises from pleiotropic effects of common QTL affecting both traits and/or from linkage disequilibrium between QTL affecting different traits. In our simulation study, the genetic correlation between traits resulted mainly from pleiotropic effects of common QTL. The results from the analysis of the simulated data indicated that, the larger the genetic correlation was, the more benefit would be obtained from a joint analysis (Table 2). However, in the case of a zero genetic correlation, the accuracies of joint genomic prediction were lower than that from separate single trait analysis for both traits. This phenomenon was also reported by Jia and Jannink[11] who observed that two continuous trait model performed worse than single trait model if no genetic correlation existed between the two continuous traits. The reason may be that, in such situation, sampling from multiple trait model leads to nonzero estimates of correlation, and then to erroneous information sharing across traits.

In the simulation study, no benefit was obtained from the joint analysis for the continuous trait in most scenarios considered. One reason is that the threshold trait with low heritability of 0.1 cannot provide enough information to help improving the accuracy of the continuous trait. Similar results were also obtained in other researches on continuous traits[15,16]. Jia and Jannink[11] simulated two continuous traits with heritability of 0.1 and 0.5, respectively, and the results showed that no increase in the accuracy for the trait with heritability of 0.5, while significant improvement was obtained for the trait with heritability of 0.1. In the present study, in the analysis of the scenario of $h_{A}^{2}=0.3$ and $h_{B}^{2}=0.5$ and of the common data set of the 14^th^ QTL-MAS workshop, where both traits had a heritability of around 0.5, improvement in accuracy were obtained for both the continuous and the threshold trait from the joint analysis using LT-BayesCπ in comparison with single trait analysis, indicating that the proposed method has the potential to improve the accuracy of genomic prediction for a continuous trait, in addition to improving the accuracy for the threshold trait with a high heritability.

It has been generally accepted that the number of QTL controlling traits of interest affects the accuracy of genomic prediction by Bayesian methods [8,11,25,26]. This was also confirmed by our results. The prediction accuracies of all methods declined with an increase in the number of QTL. When the number of QTL increases, the effect of a QTL on average should become smaller, given a fixed total genetic variance, which will also decrease the accuracy of estimating SNP effects in a given training population.

When the heritability of the continuous trait increased, the accuracies of LT-BayesCπ increased not only for the continuous trait as expected, but also for the threshold trait. Meanwhile, when the heritability of the threshold trait increased, the accuracies of LT-BayesCπ increased not only for thethreshold trait as expected, but also for the continuous trait (Fig 3). These results imply that low-heritability traits can borrow information from correlated high-heritability traits, and consequently, achieve higher prediction accuracy as also observed by Jia and Jannink[11] and Guo et al.[12]. This is also in accordance with the findings in traditional genetic evaluation that the benefit of using a multiple-trait model will be more profound for traits with lower heritability.

The accuracy for the threshold trait increased as the incidence of the threshold trait approached to 0.5. The reason is that traits with a small incidence need larger training populations to estimate variance components and thus to achieve sufficient accuracies of GEBVs [8,27]. On the other hand, the accuracy of genomic prediction for the continuous trait was not affected by the change of the incidence of the threshold trait (Fig 4). This might be due to that the variance-covariance matrix did not change in LT-BayesCπ, resulting in negligible influence for the continuous trait.

In the analysis of the simulated data, we set the values of the hyper-parameters *v*_*g*_, **V**_g_, *v*_*e*_ and **V**_e_ of the prior distributions of **G**_0_ and **R**_e_ to be -3, **0**, -3, and **0**, respectively, such that the priors reduced to flat priors. This may have an impact on the biased estimates. An alternative way to define them is to draw them from a flat distribution or other particular distributions. We will evaluate this to see their influence on the estimates of parameters in the near future.

## Conclusions

Our work indicates that the linear-threshold model based method LT-BayesCπ is useful for predicting GEBVs of a continuous and a threshold trait jointly. In particular, a joint analysis using LT-BayesCπ significantly improved the accuracy for the threshold trait compared with single trait analysis. The larger the genetic correlation between the two traits is, the more benefit would be obtained. Increasing the heritability of the continuous or/and the threshold traits is helpful to improve the genomic accuracy for both traits, particularly for the threshold trait. The incidence of the threshold trait affected the prediction accuracy only for the threshold trait. LT-BayesCπ could be a method of choice for a joint analysis of a continuous and a threshold trait.

## Supporting information

S1 Appendix The simulation data (one replication for one case) and compiled programs. (BZ)
